# Why Is ophthalmology so brilliant?

**DOI:** 10.1038/s41433-023-02560-6

**Published:** 2023-05-03

**Authors:** Rishikesh Gandhewar

**Affiliations:** Imperial College London, Faculty of Medicine, London, UK

**Keywords:** Health occupations, Education

## Introduction

Ophthalmology is a field solely dedicated towards treating disorders of the eye and visual pathway. The question arises, how can a specialty focused on an organ measuring just an inch and weighing only several grams be considered “*brilliant*”? The answer becomes clearer as we consider the significance of vision and the management of ophthalmic disease.

Our dependence on vision makes any threat to our eyes disturbing and transformative. Consequently, the ability to restore vision is a gratifying privilege shared amongst ophthalmologists. Whilst limited in organ size, ophthalmology encompasses both medicine and surgery, has roughly nine distinctive (yet interlinked) sub-specialities and offers prospects within public health and research. The breadth of opportunity presents clinicians with a balanced profession that is stimulating and ever evolving.

Furthermore, we must also consider what constitutes *brilliance*. From its French origins, brilliance refers to an object that shines brightly. Fittingly, ophthalmology is a field built upon illumination, lasers and imaging. However, to be brilliant also means to be special, skilled and clever. Therefore, we must holistically consider brilliance through multiple lenses. Firstly, the brilliance of the eye and the impact of pathology; secondly, the brilliance of the ophthalmologist; and finally, the brilliance of ophthalmic innovation.

## Vision, society and pathology

We must start by exploring sight and the impact of visual impairment. Across aeons of evolution, the eye appeared in a blink, several hundred million years ago, yet this profound instance transformed nature entirely. Its stunning intricacies became a fundamental argument for creationism, characterised by William Paisley’s infamous *watchmaker* analogy and even Charles Darwin conceded it “absurd” to consider the eye a product of evolution (although this was expertly deconstructed in his *On the Origin of Species*). Whilst initially a selective advantage in the prey-predator contest, eyes later held pervasive significance across history. In ancient Egypt, the Eye of Horus was an omnipotent symbol of well-being, healing, and protection. In the sacred tale, Horus offered his healed eye to his father, Osiris, to sustain him in the afterlife. In Hindu mythology, Shiva’s third eye, if opened, is considered apocalyptic and in *Genesis* after Adam and Eve eat the forbidden fruit “the eyes of both of them were opened”.

Today, despite reduced symbolism, vision remains integral to social functioning. A recent study discovered 88% of participants adjudged sight to be the most valuable sense [1] and yet its true value may only be appreciated during disease. Those with major visual impairments have their quality of life reduced and independence endangered. As William Shakespeare’s *Romeo* perfectly described: *“He that is strucken blind cannot forget. The precious treasure of his eyesight lost.”* Patients become unable to enjoy quotidian details whilst becoming reliant on societal adjustments, with a recent meta-analysis determining depression to be roughly 25% prevalent among ophthalmology patients [2]. Fortunately, however, many causes of visual impairment can be treated, and the return of vision can be just as transformative as its loss. This capability and privilege falls upon the field of ophthalmology. Cataract surgery typifies this notion. An operation lasting minutes can eliminate visual obscuration and re-establish 6/6 vision. This direct gratification felt in restoring vision is difficult to rival from other walks of life. Ophthalmology is brilliant because it dedicates itself to restoring vision, an ability of intrinsic value.

## The ophthalmologist

Alongside vision and pathology, the perspective of the doctor is equally important. Is ophthalmology brilliant because its doctors are? Brilliant is to be skilled and intelligent. Whilst some may consider this a pre-requisite to being a doctor, if extrapolated to mean reaching one’s potential, ophthalmology provides a vast and varied opportunity to accomplish this. Retinopathy of prematurity affects those taking their first breaths, whilst age-related macular degeneration manifests in our last decades. Acute angle-closure glaucoma is a medical emergency, whilst diabetic retinopathy runs an insidious course. Ptosis repair requires the precision of a plastic surgeon, whilst Charles Bonnet syndrome necessitates the insight of a neurologist. Ophthalmology allows each clinician to discover their niche, maximise their talent and achieve brilliance.

Furthermore, ophthalmic burden is substantial. In 2020, ophthalmology accounted for 7.9 million NHS appointments, consisting of 40% of outpatient service and major day-case operating [3]. This colossal demand is only dwarfed when considering resource-scarce regions, such as Sub-Saharan Africa, which has an estimated 2.5 ophthalmologists per million people [4]. Such demand commands opportunity for making profound impact through initiatives such as *Unite for Sight*, a charity that has cared for nearly three million patients in poverty. Moreover, this pressure has selected ophthalmology as a pioneer to areas such as Tele-medicine. We have seen development of apps, such as *Alleye*, that permit home-monitoring of macular diseases, and success of consultation platforms, such as *AttendAnywhere*, which helped sustain services through the Covid-19 pandemic [5, 6]. Ophthalmology is a richly diverse profession that allows clinicians to become well-rounded yet specialist and ultimately achieve *brilliance*.

## Ophthalmic innovation

The eye, unique in its function, is appropriately unique in its structure. This novelty has permitted multiple innovations, exemplified by the fields of gene therapy and artificial intelligence (AI). In 2017, the first gene therapy was approved for Leber congenital amaurosis, a retinal dystrophy [7]. This success is partly attributed to the immunological privilege of the eye. The eye limits the reach of the immune system in order to prioritise vision. Consequently, transplanted cells are able to avoid rejection and cell function can be rescued.

Furthermore, the transparent window created by the pupil, lens, aqueous and vitreous permits light to reach the neurosensory retina. This also allows for direct visualisation of internal tissue and AI, specifically machine-learning, has capitalised on this. A pre-requisite to building machine-learning algorithms is a substantial dataset, which materialised as 14,884 OCT scans during the Google DeepMind and Moorfields Eye Hospital collaboration. These provided high-volume, non-invasive, and high-resolution data for the software to learn from. The resultant AI system enabled instantaneous detection and prediction that rivalled expert performance in identifying retinal pathology [8]. The hope is that such clinical support systems can help ease the significant burden described previously. These brilliant innovations offer promise for efficient management across medicine.

## Conclusion

Ophthalmology, through the eyes of a foundation doctor, appears to truly be a brilliant speciality that offers an abundance of challenges, opportunities and satisfaction for both its service-users and service-providers. The importance of sight cannot be overstated, and this provides doctors with an intensely gratifying opportunity to alleviate disease and improve quality of life. The immense and intricate disease spectrum renders ophthalmology a field with ample opportunity to develop specialist aptitude.
