# Correction: Why is ophthalmology so brilliant?

**DOI:** 10.1038/s41433-023-02664-z

**Published:** 2023-07-21

**Authors:** Rishikesh Gandhewar

**Affiliations:** Imperial College London, Faculty of Medicine, London, UK

**Keywords:** Health occupations, Education

Correction to: *Eye* 10.1038/s41433-023-02560-6, published online 03 May 2023

The name ‘William Paley’ was incorrectly given as ‘William Paisley’ within the original text. The correct sentence should read, “Its stunning intricacies became a fundamental argument for creationism, characterised by William Paley’s infamous watchmaker analogy and even Charles Darwin conceded it “absurd” to consider the eye a product of evolution (although this was expertly deconstructed in his On the Origin of Species).”

The original article has been corrected.

